# Time Prediction Models for Echinococcosis Based on Gray System Theory and Epidemic Dynamics

**DOI:** 10.3390/ijerph14030262

**Published:** 2017-03-04

**Authors:** Liping Zhang, Li Wang, Yanling Zheng, Kai Wang, Xueliang Zhang, Yujian Zheng

**Affiliations:** 1 College of Medical Engineering and Technology, Xinjiang Medical University, Urumqi 830011, China; zhanglp1219@163.com (L.Z.); zhengyl_math@sina.cn (Y.Z.); wangkaimath@sina.com (K.W.); shuxue2456@126.com (X.Z.); 2 College of Public Health, Xinjiang Medical University, Urumqi 830011, China; Liwang@xjmu.edu.cn

**Keywords:** echinococcosis, grey system theory, grey forecasting model, dynamic epidemic model

## Abstract

Echinococcosis, which can seriously harm human health and animal husbandry production, has become an endemic in the Xinjiang Uygur Autonomous Region of China. In order to explore an effective human Echinococcosis forecasting model in Xinjiang, three grey models, namely, the traditional grey GM(1,1) model, the Grey-Periodic Extensional Combinatorial Model (PECGM(1,1)), and the Modified Grey Model using Fourier Series (FGM(1,1)), in addition to a multiplicative seasonal ARIMA(1,0,1)(1,1,0)_4_ model, are applied in this study for short-term predictions. The accuracy of the different grey models is also investigated. The simulation results show that the FGM(1,1) model has a higher performance ability, not only for model fitting, but also for forecasting. Furthermore, considering the stability and the modeling precision in the long run, a dynamic epidemic prediction model based on the transmission mechanism of Echinococcosis is also established for long-term predictions. Results demonstrate that the dynamic epidemic prediction model is capable of identifying the future tendency. The number of human Echinococcosis cases will increase steadily over the next 25 years, reaching a peak of about 1250 cases, before eventually witnessing a slow decline, until it finally ends.

## 1. Introduction

Echinococcosis is a globally distributed parasitic infection of humans and livestock. It can threaten human health and seriously hinder the development of animal husbandry. The worldwide prevalence of cystic Echinococcosis has fallen dramatically over the past several decades. However, Echinococcosis has increased dramatically in the Xinjiang Uygur Autonomous Region of China in the last 10 years, partially due to the nomadic nature of some minorities, the poor understanding of the transmission dynamics, and the lack of effective disease control measures.

Between 2004 and 2010, a total of 4768 human Echinococcosis cases were reported in Xinjiang, accounting for 32.55% of the total number of cases in China [1]. Data presented at the Xinjiang Center for Disease Control and Prevention (Xinjiang CDC) indicated that 2843 new cases were found in Xinjiang between 2011 and 2012. Echinococcosis continues to be a substantial cause of morbidity and mortality in Xinjiang. The large number of published reports on various aspects of Echinococcosis in Xinjiang clearly suggests that the disease is of major public health concern. At present, infection with Echinococcus remains a major public health issue, and it is regarded as one of the most serious endemic diseases in Xinjiang.

To understand the epidemic spreading phenomena of Echinococcosis and the designing of appropriate strategies to control infections, many scholars have studied different aspects of Echinococcosis [2,3,4,5,6,7]. To obtain a reliable forecast, certain laws governing the phenomena of system development must be discovered on the basis of either natural principles or real observation.

Traditional statistical model- and artificial intelligence-based approaches are the two main techniques for time series forecasting [8]. In these approaches, the predictor is built on the assumption of realizing the structure of the system to be forecasted. However, because of the limitation of information and knowledge, only part of the system’s structure can be fully realized. Grey theory was first proposed by Professor Deng [9], mainly for a system with incomplete or uncertain information, in order to construct a grey model for forecasting and decision-making. As an emerging multiple attribute, decision-making tool, which requires a limited knowledge and understanding of an unascertained system to solve problems, producing good estimations or predictions, the grey theory has caught the attention of scholars and practitioners from various disciplines and scientific fields.

The grey model, which is an effective method for modeling and forecasting small sample time series, is one of the best features of grey system theory. Generally, the grey model is written as GM(*m*,*n*), where *m* is the order and *n* is the number of variables of the modeling equation. The first-order one-variable grey model, GM(1,1), is the most widely used, and is successfully demonstrated in many applications. The GM(1,1) model is a special modeling approach based on grey exponential law, resulting from accumulated generation operations. It has an ideal fitting prediction effect for the data sequence, with homogeneous exponential characteristics. Compared with the classical statistical model and neural network model, the grey model needs a smaller sample size and data distribution is not necessary.

In recent years, the grey prediction model has been successfully utilized in many fields and has demonstrated satisfactory results [10]. Wang et al. used the grey prediction model to predict the energy consumption of a building’s heat-moisture system, and the accuracy of the prediction was high [11]. Lei et al. proposed a novel grey model PGM(1,2,a,b) to predict the price of electricity [12]. Dong et al. presented a hybrid prognosis approach, combining the age-dependent hidden Markov model (HMM) and the grey model, for prediction of engineering asset health [13]. Huang et al. created an automatic stock market forecasting and portfolio selection mechanism, based on the moving average autoregressive exogenous (ARX) prediction model, combined with grey system theory and rough set (RS) theory [14]. In these studies, it is seen that the grey prediction models can obtain satisfactory results for short-term predictions, and that grey predictors are more robust with respect to noise and to the lack of modeling information, when compared to conventional methods [12].

Using information on epidemic dynamics is also an important method of studying the spread of infectious disease, both qualitatively and quantitatively. It is based on the specific property of population growth, the spread of infectious diseases, and the related social factors, etc. It is used to construct mathematical models reflecting the dynamic properties of infectious diseases, to analyze these properties, to analyze the dynamic behavior of infectious diseases, and to pursue further simulations [15]. It is an important method for understanding the prevalence and distribution of a species, together with the factors that determine their incidence, spread, and persistence. In contrast with classic biometrics, dynamic methods can slow the transmission rules of infectious diseases from the mechanism of transmission of the disease, so that people can understand the global dynamic behavior of the transmission process. The research results are helpful for predicting the developing tendency of the infectious disease, to determine the key factors of the spread of infectious disease, and to seek the optimum strategies for preventing and controlling the spread of infectious diseases.

The traditional GM(1,1) is easy to understand and simple to calculate, with a satisfactory accuracy, but it also lacks the flexibility for adjusting the model, in order to acquire a higher forecasting precision. To improve the feasibility and effectiveness of GM(1,1), researchers have proposed many new high precision combinational prediction models, by combining different theories with grey theory [13,14]. These practical results show that the combinational prediction methods have a higher forecasting accuracy than the GM(1,1) method. In this study, the autoregressive process is combined with the grey system theory (Grey-Periodic Extensional Combinatorial Model, PECGM(1,1)), to create a human Echinococcosis forecasting model. A residual correction model based on Fourier (FGM(1,1)) is also presented for short-term forecasting. In order to verify the suitability of the FGM(1,1) model for forecasting the number of Echinococcosis cases in the short-term, a seasonal time series ARIMA model (ARIMA(1,0,1)(1,1,0)_4_) is also established for comparison. Furthermore, an epidemic dynamic model is built to evaluate the long-term trend of human Echinococcosis cases. The results of the grey predicting models show that the human Echinococcosis cases will increase in the short-term. However, the approximate estimated value of the basic reproduction number $R_{0}=0.541$ in the epidemic dynamic model, indicates that, with the current control measures, human Echinococcosis cases will decrease in the future and eventually die out.

The remainder of this paper is organized as follows. In Section 2, the methods and theories are briefly introduced. The simulation results of the different models are carefully investigated in Section 3. Finally, conclusions are made in Section 4.

## 2. Materials and Methods

### 2.1. Short-Term Prediction Models

#### 2.1.1. Original GM(1,1) Model

Although various types of grey models can be mentioned, most of the previous researchers have focused their attention on GM(1,1) models in their predictions, because of its computational efficiency. One can create a grey model to describe the behavior of a few outputs by using fewer (at least four) data points, and by using predictions to analyze future situations. Three basic operations, including accumulated generation, inverse accumulated generation, and grey modeling, are used to build a grey forecasting model. The use of an accumulated generating operator, which plays an extremely important role in grey system theory, is a method employed to make a grey process turn white. Through accumulation, one can potentially uncover a development tendency, so that the characteristics and laws of integration hidden in the chaotic original data can be sufficiently revealed.

The whitening differential equation of the GM(1,1) is a first-order differential equation. It can be established on the basis of a monotonically increasing series $X^{\left(1\right)}$, as follows:

(1) $$\frac{dx^{\left(1\right)}}{dt}+ax^{\left(1\right)}=b$$

where, a is the development coefficient, which reflects the development trend of the original series; b is the driving coefficient, which reflects the changes in the relationships between system data. a and b can be obtained by the least squares estimation.

The time response function of GM(1,1) is as follows:

(2) $${\hat{x}}^{\left(1\right)}(k+1)=(x^{\left(0\right)}(1)-\frac{b}{a})e^{-ak}+\frac{b}{a};\;k=1,2,\cdots ,n$$

The simulation and forecasting values of GM(1,1) can be evaluated as follows:

(3) $${\hat{x}}^{\left(0\right)}(k+1)={\hat{x}}^{\left(1\right)}(k+1)-{\hat{x}}^{\left(1\right)}(k)=(1-e^{a})(x^{\left(0\right)}(1)-\frac{b}{a})e^{-ak}$$

The procedures involved when using the GM(1,1) model are given in the first part of Supplementary Materials.

The GM(1,1) model hads been widely adopted in many fields. However, the effectiveness of the residual series of GM(1,1) depends on the number of data points with the same sign, which is generally low when the observations are few [16,17,18,19]. To make the grey forecasting model more adaptive and precise, some studies have developed improved residual sign estimators for the GM(1,1) model. Many scholars have worked diligently, to propose hybrid grey models that incorporate the grey prediction theory and theories or discourses in other fields of algorithm, in order to enhance the forecasting precision [14,20,21,22,23,24,25,26,27]. The above research results play an important role in promoting the development of grey system theory.

To reduce the periodic and stochastic residual errors of GM(1,1), we establish two residual error modification models, i.e., the Grey-Periodic Extensional Combinatorial Model (PECGM(1,1)) and the Modified Grey Model Using Fourier Series (FGM(1,1)), respectively.

#### 2.1.2. Grey-Periodic Extensional Combinatorial Model (PECGM(1,1))

The Grey-Periodic Extensional Combinatorial Model can be modeled as:

(4) $${\hat{x}}_{2}^{\left(0\right)}(k)={\hat{x}}_{1}^{\left(0\right)}(k)+{\hat{\varepsilon }}_{1}^{\left(0\right)}(k)+{\hat{\varepsilon }}_{2}^{\left(0\right)}(k)$$

where ${\hat{X}}^{\left(0\right)}=({\hat{x}}^{\left(0\right)}(1),{\hat{x}}^{\left(0\right)}(2),\cdots ,{\hat{x}}^{\left(0\right)}(n))$ is the fitting sequence of GM(1,1),

$${\hat{x}}_{1}^{\left(0\right)}(k)=(1-e^{a})[x^{\left(0\right)}(1)-\frac{b}{a}]e^{-a(k-1)};\;k=1,2,\cdots ,n.$$

${\hat{E}}_{1}=({\hat{\varepsilon }}_{1}(1),{\hat{\varepsilon }}_{1}(2),\cdots ,{\hat{\varepsilon }}_{1}(n))$ is the fitting sequence of one-time residual sequence, and ${\hat{\varepsilon }}_{1}^{\left(0\right)}(k)$ is the sum of values at the same moment of different preferred periods,

(5) $${\hat{\varepsilon }}_{1}^{\left(0\right)}(k)=\sum_{l\in I}f_{l}(k)$$

${\hat{E}}_{2}=({\hat{\varepsilon }}_{2}(1),{\hat{\varepsilon }}_{2}(2),\cdots ,{\hat{\varepsilon }}_{2}(n))$ is the simulate value of the two-time residual sequence by an ARIMA time series model. The procedures involved for using the PECGM(1,1) model are given in the second part of Supplementary Materials.

#### 2.1.3. Modified Grey Model Using Fourier Series (FGM(1,1))

Besides the variation tendency, the tendency for periodic and stochastic phenomena also commonly exist in time series. The forecasting precision of the GM(1,1) model for a data sequence with large random fluctuations, is always low. In this study, the Fourier series is always adopted to improve the prediction performance of the original grey model [28].

Assume $X^{\left(0\right)}=(x^{\left(0\right)}(1),x^{\left(0\right)}(2),\cdots ,x^{\left(0\right)}(n))$ is a non-negative time sequence. A Fourier series correction can be obtained as follows:

(6) $${\hat{x}}_{2}^{\left(0\right)}(k)={\hat{x}}_{1}^{\left(0\right)}(k)+{\hat{\varepsilon }}^{\left(0\right)}(k),\;k=2,3,\cdots ,n.$$

(7) $${\hat{\varepsilon }}_{1}^{\left(0\right)}(k)≅\frac{1}{2}a_{0}+\sum_{i=1}^{m}\left[a_{i}\cos(\frac{2\pi i}{T}k\right)+b_{i}\sin(\frac{2\pi i}{T}k)];\;k=2,3,\cdots ,n,$$

where ${\hat{X}}^{\left(0\right)}=({\hat{x}}^{\left(0\right)}(1),{\hat{x}}^{\left(0\right)}(2),\cdots ,{\hat{x}}^{\left(0\right)}(n))$ is the fitting sequence of GM(1,1),

(8) $${\hat{x}}_{1}^{\left(0\right)}(k)=(1-e^{a})[x^{\left(0\right)}(1)-\frac{b}{a}]e^{-a(k-1)};\;k=1,2,\cdots ,n.$$

The procedures involved when using the FGM(1,1) model are also given in the third part of Supplementary Materials.

The PECGM(1,1) and the FGM(1,1) are two error-modified grey models in which the residual is adjusted by GM(1,1). The grey-periodic extensional combinatorial model belongs to an error periodic extensional model based on GM(1,1), with a preferred period to contrast new data sequences and to add the values at the same moment of different periods. In the FGM(1,1) model, the Fourier series is employed to modify the implied periodic phenomena in the one-time residual series. Note that the Fourier series is only used to increase the prediction capability from the considered input data set, and thus, does not change the local characteristics of GM(1,1). These two models overcome the unpredictability due to the monotonicity and periodical fluctuation of the time series.

#### 2.1.4. The ARIMA Model

The ARIMA model is one of the most popular univariate time series models, which is widely used in infectious disease modeling [29,30,31]. The ARIMA model is based on the ARMA models, linearly expressing the current human Echinococcosis cases with its previous cases (autoregressive) and the residual series (moving average). The ARMA model can be expressed as:

(9) $$y(t)=\phi_{1}y(t-1)+\cdots +\phi_{p}y(t-p)+\varepsilon (t)-\theta_{1}\varepsilon (t-1)-\cdots -\theta_{q}(t-q)$$

where y(t) refers to the value of the time series at time t and ε(t) is the residual at time t. ϕ and θ are their corresponding coefficients, respectively.

The ARIMA model deals with a non-stationary time series, using a differencing process based on the ARMA model. The model can be expressed as ARIMA (*p*,*d*,*q*) × (*P*,*D*,*Q*)*_s_*, where *p*, *d*, and *q* are non-negative integers, which refer to the order of the autoregressive, integrated, and moving average parts of the model, respectively; whereas *P*, *D*, and *Q* represent the order of the seasonal autoregressive, differencing, and moving average, respectively (not shown in the above equation). The subscripted letter “*s*” shows the seasonal period length. The ARIMA modeling procedure introduced by Box and Jenkins, consists of three iterative steps: identification, estimation, and diagnostic checking [32]. To test the validity of FGM(1,1), an autoregressive integrated moving average is also used to fit the univariate time series model of human Echinococcosis cases in Xinjiang.

#### 2.1.5. Model Accuracy Examination

To compare the accuracy of the proposed forecasting models, three evaluation criteria are used here: the mean absolute deviation (MAD), the mean absolute percentage error (MAPE), and the root mean square error (RMSE). They are expressed as follows:

(10) $$\mathrm{MAD}=\frac{\sum_{i=1}^{n}\left|x^{\left(0\right)}(k)-{\hat{x}}^{\left(0\right)}(k)\right|}{n}$$

(11) $$\mathrm{MAPE}=(\frac{1}{n}\sum_{k=1}^{n}\left|\frac{x^{\left(0\right)}(k)-{\hat{x}}^{\left(0\right)}(k)}{x^{\left(0\right)}(k)}\right|)\times 100\%$$

(12) $$\mathrm{RMSE}=\sqrt{\frac{\sum_{k=1}^{n}{\left[(x^{\left(0\right)}(k)-{\hat{x}}^{\left(0\right)}(k))\right]}^{2}}{n}}$$

where $x^{\left(0\right)}(k)$ is the actual value at time k, ${\hat{x}}^{\left(0\right)}(k)$ is its fitting value, and n is the number of data points used for the prediction.

### 2.2. Long-Term Prediction Model

The grey models, which demonstrate an optimal and unique ability for performing fitting predictions using small data sets and limited information, often obtain a higher precision in short-term predicting. However, because of the inherent bias in the traditional grey-forecasting FGM(1,1) model and the fixed choice of its parameter, the forecast precision is relatively low and unsuitable for long-term forecasting. To catch the long-term tendency of a time series, the transmission mechanism implied in the time series must be considered. The epidemic dynamic model, based on the transmission mechanism of Echinococcosis, is adapted in this study after Wang et al. [1], and is described below:

(13) $$\{\begin{array}{l} \frac{dS_{D}}{dt}=A_{1}-\beta_{1}S_{D}I_{L}-d_{1}S_{D}+\sigma I_{D}\\ \frac{dI_{D}}{dt}=\beta_{1}S_{D}I_{L}-(d_{1}+\sigma )I_{D},\\ \frac{dS_{L}}{dt}=A_{2}-\beta_{2}S_{L}x-d_{2}S_{L},\\ \frac{dI_{L}}{dt}=\beta_{2}S_{L}x-d_{2}I_{L},\\ \frac{dx}{dt}=aI_{D}-dx,\\ \frac{dS_{H}}{dt}=A_{3}-\beta_{3}S_{H}x-d_{3}S_{H}+\gamma I_{H},\\ \frac{dE_{H}}{dt}=\beta_{3}S_{H}x-(d_{3}+\omega )E_{H},\\ \frac{dI_{H}}{dt}=\omega E_{H}-(d_{3}+\mu +\gamma )I_{H}. \end{array}$$

For the dog population, assume the annual recruitment rate and the natural death rate are $A_{1}$ and $d_{1}$, respectively; The recovery rate of transition from infected to non-infected dogs, including the natural recovery rate and recovery due to anthelmintic treatment, is σ; let $\beta_{1}S_{D}I_{L}$ describe the transmission of Echinococcosis between susceptible dogs and infectious livestock after the ingestion of cyst-containing organs of the infected livestock. For the livestock population, suppose the annual recruitment rate is $A_{2}$ and the death rate is $d_{2}$; the transmission of Echinococcosis to livestock by the ingestion of Echinococcus eggs in the environment is described as $\beta_{2}S_{L}x$. For Echinococcus eggs, assume the released rate from infected dogs is a and the mortality rate is d. For a human population, suppose the annual recruitment rate is $A_{3}$, the natural death rate is $d_{3}$, and the disease-related death rate is μ; the incubation period of infected individuals is denoted by 1/ω and the recovery rate is denoted by γ. The transmission of Echinococcosis to the human population by the ingestion of Echinococcus eggs in the environment is described as $\beta_{3}S_{H}x$. All parameters are assumed to be positive.

The population of dogs is divided into two classes: the susceptible population and the infected population, denoted by $S_{D}(t)$ and $I_{D}(t)$, respectively. The livestock population is divided into two classes: susceptible and infectious, denoted by $S_{L}(t)$ and $I_{L}(t)$, respectively The density of Echinococcus eggs is denoted by x(t). The human population is divided into three classes: susceptible, exposed, and infectious, denoted by $S_{H}(t)$, $E_{H}(t)$, and $I_{H}(t)$, respectively.

The basic reproductive number $R_{0}$ is defined as:

(14) $$R_{0}=\sqrt[{3}]{\frac{\beta_{1}\beta_{2}A_{1}A_{2}a}{(d_{1}+\sigma )d_{1}d_{2}^{2}d}}$$

The basic reproductive number $R_{0}$ determines the dynamics of the model [1]. The disease-free equilibrium is globally asymptotically stable when $R_{0}<1$. The disease-free equilibrium is unstable and the endemic equilibrium is globally asymptotically stable if $R_{0}>1$.

## 3. Results

### 3.1. Short-Term Prediction Results

Data on the epidemiological cases in humans were obtained from the quarterly report of Xinjiang CDC. The 33 observations from the first quarter of 2004 (2004(Q1)) to the first quarter of 2012 (2012(Q1)), are used to establish the fitting models and the remaining observations from the second quarter (2012(Q2)) to the fourth quarter (2012(Q4)), which are utilized as test objects to compare their performance. In this section, to present a short-term prediction of the Echinococcosis transmission in Xinjiang, the GM(1,1), PECGM(1,1), FGM(1,1), and seasonal time series model ARIMA(1,0,1)(1,1,0)_4_, are established for comparison. The results are listed in Table 1.

Table 1 demonstrates that the performance of FGM(1,1) is better than GM(1,1), PECGM(1,1), and ARIMA(1,0,1)(1,1,0)_4_, based on the MAD, the MAPE, and the RMSE.

**Figure 1:**
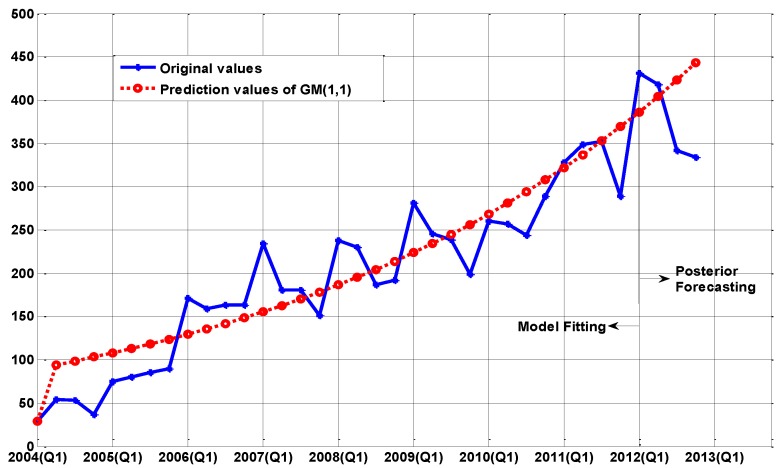
The comparison between the real values and the simulation values of the GM(1,1) model.

**Figure 2:**
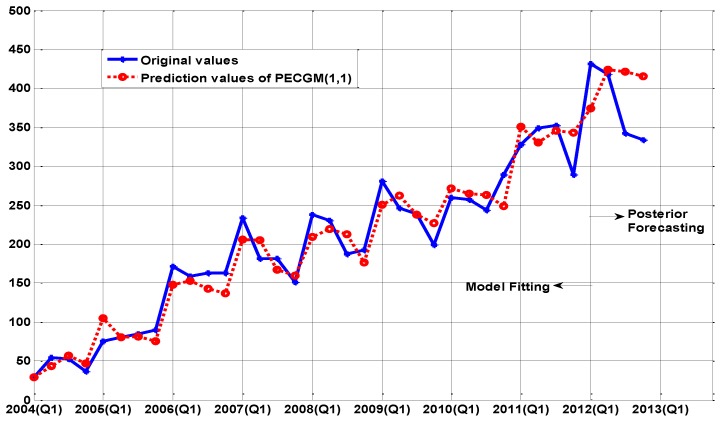
The comparison between the real values and the simulation values of the PECGM(1,1) model.

**Figure 3:**
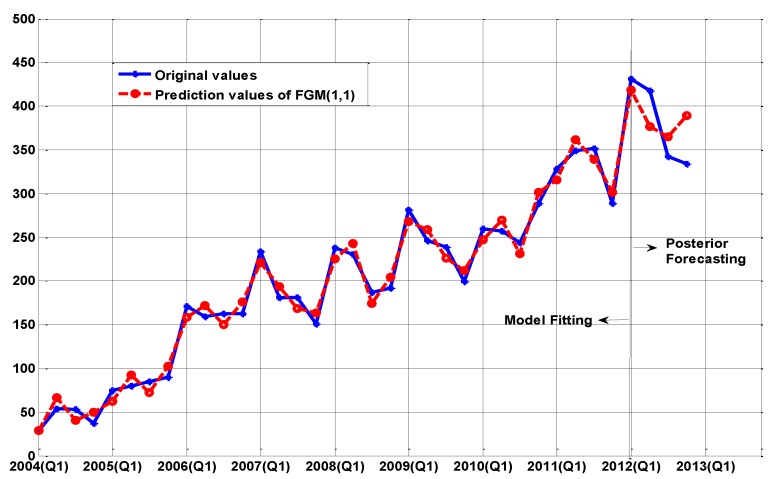
The comparison between the real values and the simulation values of the FGM(1,1) model.

The fitting curves are shown in Figure 1, Figure 2 and Figure 3, respectively. For short-term forecasting, the results clearly indicate that the values forecasted by these models are very close to the real values.

As we can see from Table 1 and Figure 1, the traditional GM(1,1) model exhibits a better performance in trend prediction. However, the GM(1,1) model has over-shooting phenomena on the change-points, and further causes the delay effects among the whole prediction line, so the prediction precision is not very satisfactory.

**Figure 4:**
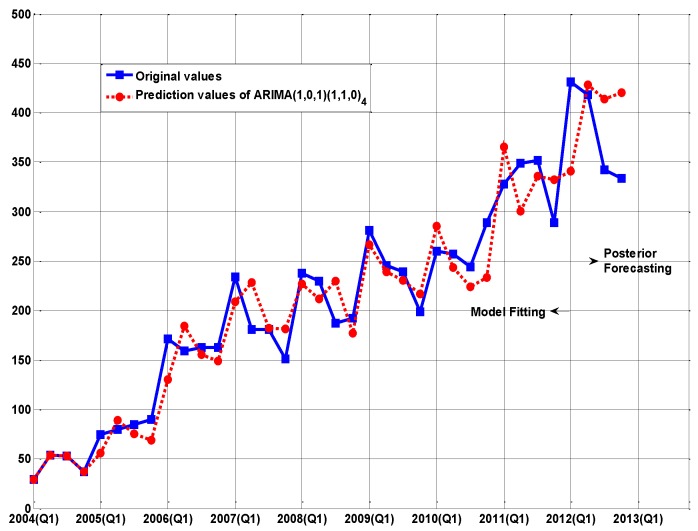
The comparison between the real values and the simulation values of the ARIMA(1,0,1)(1,1,0)_4_ model.

Figure 2, Figure 3 and Figure 4 demonstrate the other three models (PECGM(1,1), FGM(1,1), and ARIMA(1,0,1)(1,1,0)_4_ models), which demonstrate a better adaptability to structural change than the GM(1,1) model. Fitting curves show that the prediction results of the PECGM(1,1), FGM(1,1), and ARIMA(1,0,1)(1,1,0)_4_ models can synchronously vibrate with the actual curve, and there are obvious seasonal and periodic features in Echinococcosis cases in Xinjiang; for example, spring is the epidemic season for Echinococcosis. The fitting results of the four models shown in Table 1 indicate that, using present control methods, the number of Echinococcosis cases will increase in the short-term.

Table 1 and Figure 3 illustrate that the prediction accuracy of the FGM(1,1) model for 2012(Q2)–2012(Q4) is significantly higher than the other models, and that the simulation errors from 2004(Q1) to 2012(Q1) are also notably smaller. In contrast to the PECGM(1,1) and the ARIMA(1,0,1)(1,1,0)_4_ models, the FGM(1,1) model produces better simulations and predictions. By using Fourier techniques to transform the one-time residual series into frequency spectra, the low-frequency terms can be selected and the FGM(1,1) model can filter out the noise at the high-frequency modes, to obtain better performance of the prediction. As shown in Equation (3), we note that GM(1,1) and FGM(1,1) are based on the exponential function and may have unsatisfactory results when employed in the modeling and predicting of non-smooth curves for a long period of time.

### 3.2. Long-Term Prediction Results

#### 3.2.1. Fitting Results

The prevention and treatment of Echinococcosis action plan for 2010–2015 was formulated by the Ministry of Health of the People’s Republic of China, on 24 December 2010. Recent technological advances may facilitate the implementation of improved control programmes and reduce the time frame required for elimination. We expect that this will significantly reduce the incidence of Echinococcosis infection in Xinjiang. In this section, we use the epidemic dynamic model proposed by Wang et al. [1] to give a prediction of Echinococcosis in the long-term.

We select and calculate the main model parameters by fitting the model to quarterly data of Echinococcosis [1]. The parameter list is as follows [33].

Based on the parameter values listed in Table 2, we chose the initial values of the model as follows:

(15) $$S_{D}(0)=2\times 10^{6}/4,\;I_{L}(0)=1.425\times 10^{7},\;x(0)=1.44\times 10^{7}/4,$$

(16) $$S_{H}(D)=0.29\times 10^{7},\;E_{H(0)}=625,\;I_{H}(0)=29.$$

Using these parameter values, we can give a rough estimate of the basic reproduction number $R_{0}=0.541$ in the current circumstances in Xinjiang. This indicates that, with the current control measures, human Echinococcosis cases will eventually vanish in Xinjiang.

**Figure 5:**
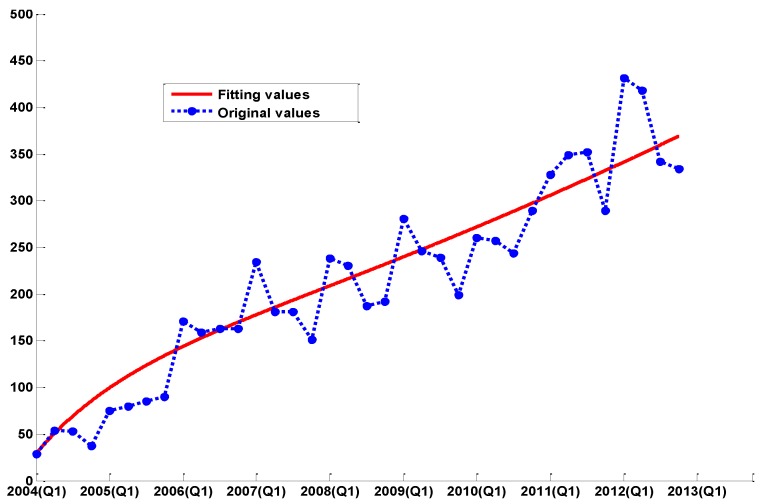
The comparison between real values and the simulation values.

The simulation results of the model on the number of human Echinococcosis cases are shown in Figure 5.

**Figure 6:**
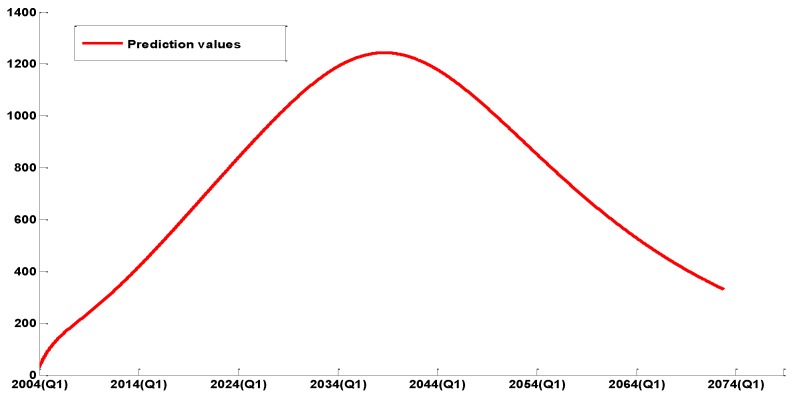
The tendency prediction of human Echinococcosis cases over a period of 60 years.

From Figure 5, we can see that the dynamic epidemic model simulation result matches the Echinococcosis data well. According to the current situation, we present a prediction on the general tendency of the epidemic in the long-term, which is presented in Figure 6.

Figure 6 shows that the number of human Echinococcosis cases will increase steadily in 26 or 27 years, then reach a peak (about 1250) in 2039, before beginning to slowly decline, and finally disappear.

#### 3.2.2. Sensitivity Analysis

To create better control strategies for Echinococcosis infections, we would like to see what parameters can reduce the basic reproduction number $R_{0}$. Based on the methods of reference [34], we performed a sensitivity analysis of several model parameters and provided some useful strategies for controlling the transmission of Echinococcosis.

**Figure 7:**
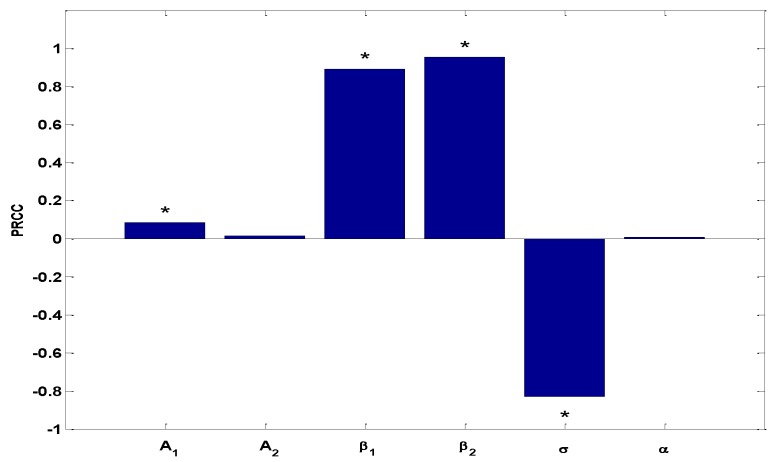
Partial rank correlation coefficients (PRCC) results for the dependence of $R_{0}$ on each parameter. * denotes the value of PRCC is not zero significantly, where the significance level is 0.05.

For the sensitivity analysis, we used Latin hypercube sampling (LHS) and partial rank correlation coefficients (PRCC) to examine parameters which had a significant influence on the basic production number $R_{0}$. We chose the sample size *n* = 3000, and the significance level α = 0.05. The larger the PRCC is in absolute value, the more important the parameters are for responding to the change of $R_{0}$. A plus sign or minus sign means that the influence is positive or negative, respectively. The ordering of these PRCCs corresponds to the level of statistical influence that the parameter has on the variability of the basic production number $R_{0}$. The PRCC values of six parameters are listed in Table 3 and are shown in Figure 7.

From Table 3 and Figure 7, we can confirm that parameters $A_{1}$, $\beta_{1}$, and $\beta_{2}$ have a positive impact upon $R_{0}$, and that σ has a negative impact. We also know that $R_{0}$ is not sensitive to parameters $A_{2}$ and α. Further, Table 3 shows that the low parasite egg-to-livestock transmission rate $\beta_{2}$ (|PRCC| = 0.9530) has the greatest impact on $R_{0}$, followed by the livestock to dog transmission rate $\beta_{1}$ (|PRCC| = 0.8916), and then the high rate moving from infected to non-infected dogs σ (|PRCC| = −0.8307). Hence, from sensitivity and mathematical analysis, we conclude that the most effective approach for reducing the Echinococcosis infection is to decrease the parameters $\beta_{1}$, and $\beta_{2}$, and to increase the parameter σ.

From the above sensitivity analysis, to control Echinococcosis, some alternative strategies can be considered: dogs should be barred from slaughter houses and should not be fed uncooked offal, infection carcasses and offal should be burned or buried, the frequency of dog anthelmintic should be increased, the method of transmission and instructions in personal sanitation should be informed to the public, and the annual crop of newborn puppies should be reduced, etc. [1].

## 4. Conclusions

Echinococcosis is of significant medical and economic importance in the Xinjiang Uygur Autonomous Region of China. It is one of the most important zoonotic diseases and it is of great social importance. This research investigates the modified grey model and the dynamic epidemic model for predicting the human Echinococcosis cases. In this study, the traditional GM(1,1) model, two residual correction-based grey models (PECGM(1,1), FGM(1,1)), and a multiplicative seasonal model ARIMA(1,0,1)(1,1,0)_4_ are applied, to analyze the surveillance data of Echinococcosis cases for a short-term prediction comparison. Furthermore, a dynamic epidemic model for long-term prediction is also established.

The fitting results of the grey models show that FGM(1,1) is able to make an accurate prediction for the Echinococcosis prevalence trend in Xinjiang. The results also demonstrate that there are obvious seasonal and periodic features in Echinococcosis cases in Xinjiang. Infection with Echinococcus remains a major public health issue and the cases will continue to rise in the short-term. Efforts should be continued, for both animals and humans, by increasing training campaigns and public awareness.

A dynamic epidemic prediction model can predict the future tendency very well. Its results demonstrate that, using current control options, human Echinococcosis cases will decrease around the 104th quarter. The basic reproduction number $R_{0}=0.541$ indicates that, with the current control measures, human Echinococcosis cases will cease to exist in Xinjiang in the long run. To control human Echinococcosis, we could choose prevention and control strategies from decresae parameters $A_{1}$ (annual crop of newborn puppies), $\beta_{1}$ (livestock to dog transmission rate), $\beta_{2}$ (parasite egg-to-livestock transmission rate), and increase the parameter σ (rate moving from infected to non-infected dog). However, elimination is a difficult goal to achieve, principally due to the disease transmission restriction and the control measures being implemented.

It should be noted that the government and officials in Xinjiang have enlarged the propaganda and education on Echinococcosis control. There are many clear technological improvements in the diagnosis and treatment of human and animal Echinococcosis vaccinations against Echinococcus granulosus in animals. These new measures and technologies increase the efficiency of Echinococcus control programmes, potentially reducing the time required for its elimination. We hope that our work may help in understanding the epidemic spreading phenomena and designing appropriate strategies to control Echinococcus infections in Xinjiang.

## Figures and Tables

**Table 1:** Forecasting results and performance evaluation of original values, GM(1,1) model, PECGM(1,1) model, FGM(1,1) model, and ARIMA(1,0,1)(1,1,0)_4_.

Time	Original Values	GM(1,1)	PECGM(1,1)	FGM(1,1)	ARIMA(1,0,1)(1,1,0)_4_
2004-Q1	29				
2004-Q2	54	94.1602	43.2064	66.6371	53.9460
2004-Q3	53	98.5481	56.6699	40.3629	52.9470
2004-Q4	37	103.1405	46.8164	49.6371	36.9630
2005-Q1	75	107.947	104.9348	62.3629	56.0506
2005-Q2	80	112.9774	80.6936	92.6371	88.8068
2005-Q3	85	118.2423	81.1441	72.3629	75.6675
2005-Q4	90	123.7525	75.6184	102.6371	68.5544
2006-Q1	171	129.5195	148.0873	158.3629	130.5286
2006-Q2	159	135.5552	152.8614	171.6371	184.2488
2006-Q3	163	141.8722	142.374	150.3629	155.4327
2006-Q4	163	148.4836	136.5795	175.6371	148.7839
2007-Q1	234	155.403	206.1309	221.3629	208.9408
2007-Q2	181	162.645	204.6812	193.6371	228.0572
2007-Q3	181	170.2244	167.3362	168.3629	182.2278
2007-Q4	151	178.157	159.3529	163.6371	181.2402
2008-Q1	238	186.4593	209.4171	225.3629	227.2463
2008-Q2	230	195.1485	219.1547	242.6371	211.5525
2008-Q3	187	204.2426	212.3443	174.3629	229.9090
2008-Q4	192	213.7605	176.2863	204.6371	177.1743
2009-Q1	281	223.7219	250.2797	268.3629	266.3309
2009-Q2	246	234.1475	261.9837	258.6371	239.4284
2009-Q3	239	245.059	237.8308	226.3629	230.8546
2009-Q4	199	256.479	226.4649	211.6371	216.9646
2010-Q1	260	268.4312	271.189	247.3629	284.9956
2010-Q2	257	280.9403	264.9865	269.6371	243.4996
2010-Q3	244	294.0324	262.9642	231.3629	223.7910
2010-Q4	289	307.7346	248.4205	301.6371	233.6549
2011-Q1	328	322.0753	350.6532	315.3629	365.3474
2011-Q2	349	337.0843	330.7005	361.6371	300.1962
2011-Q3	352	352.7928	345.6146	339.3629	335.7246
2011-Q4	289	369.2333	342.7292	301.6371	332.4649
2012-Q1	431	386.4399	374.0377	418.3629	340.5767
MAD		31.9641	19.2301	12.6371	22.9506
MAPE (%)		25.0438	10.4278	8.6256	11.7100
RMSE		37.448	23.1066	12.4442	30.2869
2012-Q2	418	404.4483	423.8045	376.9253	428.4281
2012-Q3	342	423.296	421.0678	365.1108	413.7626
2012-Q4	334	443.02	415.5579	389.5186	420.2279
MAD		67.96	47.3842	28.1548	56.1395
MAPE (%)		19.8847	16.3088	11.0688	16.4300
RMSE		78.906	65.6686	42.0458	65.0483

**Table 2:** Parameters and their values (unit: $quarter^{-1}$).

Parameters	Value	Comments
$A_{1}$	$2\times 10^{4}$	annual crop of newborn puppies
$d_{1}$	$2\times 10^{-2}$	dog natural mortality rate
$\beta_{1}$	$1\times 10^{-4}$	livestock to dog transmission rate
σ	0.5	rate moving from infected to non-infected dog
$A_{2}$	$2.625\times 10^{7}$	annual crop of newborn livestock
$d_{2}$	$8.25\times 10^{-2}$	livestock mortality rate
$\beta_{2}$	$2.4\times 10^{-13}$	parasite egg-to-livestock transmission rate
a	1.65	released rate from infected dog
d	47.5	parasite egg mortality rate
$A_{3}$	$6\times 10^{4}$	human annual birth population
$d_{3}$	$3.525\times 10^{-3}$	human natural mortality rate
ω	$3.125\times 10^{-3}$	human incubation period
μ	0.0238%	human disease-related death rate
γ	0.1875	treatment/recovery rate
$\beta_{3}$	$1.325\times 10^{-9}$	parasite egg-to-human transmission rate

**Table 3:** Partial rank correlation coefficients (PRCCs) for the aggregate $R_{0}$ and each input parameter variable.

Input Parameter	The Basic Production Number $R_{0}$	
	PRCC	p Value
$A_{1}$	0.0833	0.0000
$A_{2}$	0.0148	0.4183
$\beta_{1}$	0.8916	0.0000
$\beta_{2}$	0.9530	0.0000
σ	−0.8307	0.0000
α	0.0071	0.6964

## Supplementary Materials

The following are available online at http://www.mdpi.com/1660-4601/14/3/262/s1. 1. Original GM(1,1) Model; 2. Grey-Periodic Extensional Combinatorial Model (PECGM(1,1)); 3. Modified Grey Model Using Fourier Series (FGM(1,1)).
