# Supplementary material for: Time Prediction Models for Echinococcosis Based on Gray System Theory and Epidemic Dynamics
Source: Int J Environ Res Public Health. 2017 Mar 4;14(3):262. doi: 10.3390/ijerph14030262 (PMC5369098; doi:10.3390/ijerph14030262)
Supplement: Supplementary file 1 [file ijerph-14-00262-s001.pdf]

Supplementary Materials: Time Prediction Models for Echinococcosis Based on Gray System Theory and Epidemic Dynamics

Liping Zhang, Li Wang, Yanling Zheng, Kai Wang, Xueliang Zhang and Yujian Zheng

In this supplementary material, we provide more detailed information about the procedures of the three grey models: GM(1,1), the Grey-Periodic Extensional Combinatorial Model, (PECGM(1,1)), and the residual correction model based on Fourier (FGM(1,1)).

1. Original GM(1,1) Model

The procedures involved for using the GM(1,1) model can be summarized as follows [1–5].

Step 1: Establish the 1-AGO sequence and the generated mean original time series.

Let the original non-negative time series be $X^{(0)}=(x^{(0)}(1),x^{(0)}(2),\cdots,x^{(0)}(n))$, where $n\geq 4$. It's 1-AGO (accumulated generating operator) sequence $X^{(1)}$ is:

$$X^{(1)}=(x^{(1)}(1),x^{(1)}(2),\cdots,x^{(1)}(n)), \tag{1}$$

where:

$$x^{(1)}(k)=\sum_{i=1}^{k}x^{(0)}(i);\quad k=1,2,\cdots,n. \tag{2}$$

The generated mean sequence $Z^{(1)}$ of $X^{(1)}$ can be evaluated as:

$$Z^{(1)}=(z^{(1)}(2),z^{(1)}(3),\cdots,z^{(1)}(n)), \tag{3}$$

where:

$$z^{(1)}(k)=\frac{1}{2}(x^{(1)}(k)+x^{(1)}(k-1)),\quad k=2,3,\cdots,n. \tag{4}$$

Step 2: Define the differential equation of the GM(1,1) model.

The grey differential equation of the GM(1,1) is defined as:

$$x^{(0)}(k)+az^{(1)}(k)=b. \tag{5}$$

The whitenization differential equation of the GM(1,1) is a first-order differential equation. It can be established on the basis of monotonically increasing series $X^{(1)}$ as follows:

$$\frac{dx^{(1)}}{dt}+ax^{(1)}=b, \tag{6}$$

where a is the development coefficient, which reflects the development trend of the original series; and b is the driving coefficient, which reflects the changes in the relationship between system data.

Step 3: Estimate the model parameters a and b .

The least squares estimation of a and b can be obtained as:

$$\hat{a}=[a,b]^{T}=(B^{T}B)^{-1}B^{T}Y, \tag{7}$$

where:

$$Y=\begin{bmatrix} x^{(0)}(2) \\ x^{(0)}(3) \\ \vdots \\ x^{(0)}(n) \end{bmatrix},\quad B=\begin{bmatrix} -z^{(1)}(2) & 1 \\ -z^{(1)}(3) & 1 \\ \vdots & 1 \\ -z^{(1)}(n) & 1 \end{bmatrix} \tag{8}$$

Step 4: Establish the time response function and the foresting model.

Make $\hat{x}^{(1)}(1)=x^{(0)}(1)$, producing the time response function:

$$\hat{x}^{(1)}(k+1)=(x^{(0)}(1)-\frac{b}{a})e^{-ak}+\frac{b}{a};\quad k=1,2,\cdots,n\cdot \tag{9}$$

When applying the first-order inverse accumulation operation (1-IAGO) to $\hat{x}^{(1)}(k+1)$, we can obtain the simulation and forecasting values:

$$\hat{x}^{(0)}(k+1)=\hat{x}^{(1)}(k+1)-\hat{x}^{(1)}(k)=(1-e^{a})(x^{(0)}(1)-\frac{b}{a})e^{-ak}\cdot \tag{10}$$

2. Grey-Periodic Extensional Combinatorial Model (PECGM(1,1)) [6]

Let $X^{(0)}=(x^{(0)}(1),x^{(0)}(2),\cdots,x^{(0)}(n))$ be an original non-negative time series and n be the sample size of the data, where $n\geq 4$. The procedure for the Grey-Periodic Extensional Combinatorial Model can be explained as follows.

Step 1: Establish the GM(1,1) forecasting model of the original sequence.

Let $\hat{X}_1^{(0)}=(\hat{x}_1^{(0)}(1),\hat{x}_1^{(0)}(2),\cdots,\hat{x}_1^{(0)}(n))$ be the fitted and forecasted series of $X^{(0)}$,

$$\hat{x}_1^{(0)}(k)=(1-e^{a})[x^{(0)}(1)-\frac{b}{a}]e^{-a(k-1)};\quad k=1,2,\cdots,n. \tag{11}$$

The error sequence of $X^{(0)}$ can be defined as:

$$E_1^{(0)}=(\varepsilon_1^{(0)}(1),\varepsilon_1^{(0)}(2),\cdots,\varepsilon_1^{(0)}(n)), \tag{12}$$

where:

$$\varepsilon_1^{(0)}(k)=x^{(0)}(k)-\hat{x}_1^{(0)}(k);\quad k=1,2,\cdots,n. \tag{13}$$

Step 2: Construct the mean generating function matrix.

Define the mean generating function of $E_1^{(0)}$ by:

$$\bar{z}_m(i)=(\sum_{j=1}^{n_m}\varepsilon_1^{(0)}(i+(j-1)m))\Big/n_m;\quad i=1,2,\cdots,m,\quad 1\leq m\leq M; \tag{14}$$

where n is the the sample sequence size, $n_m=[n/m]$ and $M=\left[\frac{n}{2}\right]$.

One can establish the mean generating function matrix of $E_1^{(0)}$ as follows:

$$Z = \begin{bmatrix} \bar{z}_1(1) & \bar{z}_2(1) & \bar{z}_3(1) & \cdots & \bar{z}_M(1) \\ & \bar{z}_2(2) & \bar{z}_3(2) & \cdots & \bar{z}_M(2) \\ & & \bar{z}_3(3) & \cdots & \bar{z}_M(3) \\ & & & \ddots & \cdots \\ & & & & \bar{z}_M(M) \end{bmatrix} \tag{15}$$

Step 3: Establish the period extension function.

We use the periodic extension for $\bar{z}_m(i)$ by:

$$f_m(k) = \bar{z}_m(k); \quad k = i[\mathrm{mod}(m)]; \quad k = 1,2,\cdots,n. \tag{16}$$

Step 4: Extract the dominant period of the two-time residual sequence.

Define $S^{(m)}$ and S as:

$$S^{(m)} = \sum_{i=1}^{m} n_i(\bar{z}_m(i) - \bar{\varepsilon})^2, \tag{17}$$

$$S = \sum_{i=1}^{m}\sum_{j=1}^{n_m}(\varepsilon^{(0)}(i+(j-1)m) - \bar{z}_m(i))^2, \tag{18}$$

where:

$$n_i = n/i, \quad \bar{\varepsilon} = (\sum_{i=1}^{n}\varepsilon_1^{(0)}(i))\Big/n. \tag{19}$$

Let:

$$F^{(m)} = (n-m)S^{(m)}/(m-1)S. \tag{20}$$

Thus, $F^{(m)} \sim F(m-1, n-1)$. If $F^{(m)} > F_\alpha(m-1, n-m)$ for a given confidence level α, then $E_1^{(0)}$ contains a dominant period of length m.

Step 5: Extract the other dominant periods of the error sequence.

Let E_2 be the two-time error sequence,

$$E_2 = (\varepsilon_2(1), \varepsilon_2(2), \cdots, \varepsilon_2(n)), \tag{21}$$

where:

$$\varepsilon_2(k) = \varepsilon_1^{(0)}(k) - f_m(k); \quad k = 1,2,\cdots,n. \tag{22}$$

By repeating step 4 and step 5, the other dominant periods of $E_1^{(0)}$ can be extracted.

Let I be the set of different dominant period lengths. Define $\hat{\varepsilon}_1^{(0)}(k)$ as:

$$\hat{\varepsilon}_1^{(0)}(k) = \sum_{l \in I} f_l(k), \tag{23}$$

$\hat{\varepsilon}_1^{(0)}(k)$ is the approximate value of $\varepsilon_1^{(0)}(k)$.

Step 6: Calculate the one-time error correction values.

$$\hat{x}_2^{(0)}(k) = \hat{x}_1^{(0)}(k) - \hat{\varepsilon}_1^{(0)}(k); \quad k = 1,2,\cdots,n. \tag{24}$$

Step 7: Establish the PECGM(1,1).

Besides the presence of the periodic phenomenon, the stochastic phenomenon also commonly exists in the time series. In order to reduce the random noise and improve the accuracy of the model, an autoregressive process $AR(p)$ is used to establish a two-time residual correction model.

Assume X_t is a stationary process with zero mean, the autoregressive process is formulated as:

$$X_t = \varphi_1 X_{t-1} + \varphi_2 X_{t-2} + \cdots + \varphi_p X_{t-p} + \varepsilon_t. \tag{25}$$

We denote such a process by $AR(p)$, and p is called the order of the process; $\varphi_i (i = 1,2,\cdots,p)$ are the model parameters; ε_t is a white noise time series.

Let $E_2^{(0)}$ be the new two-time residual sequence after Step 6,

$$E_2^{(0)} = (\varepsilon_2^{(0)}(1), \varepsilon_2^{(0)}(2), \cdots, \varepsilon_2^{(0)}(n)), \tag{26}$$

where $\varepsilon_2^{(0)}(k) = x^{(0)}(k) - \hat{x}_2^{(0)}(k)$.

Denote the fitted value of $\varepsilon_2^{(0)}(k)$ by $\hat{\varepsilon}_2^{(0)}(k)$. Thus, we have:

$$\hat{x}_2^{(0)}(k) = \hat{x}_1^{(0)}(k) + \hat{\varepsilon}_1^{(0)}(k) + \hat{\varepsilon}_2^{(0)}(k). \tag{27}$$

3. Modified Grey Model Using Fourier Series (FGM(1,1))

Assume $X^{(0)} = (x^{(0)}(1), x^{(0)}(2), \cdots, x^{(0)}(n))$ is a non-negative time sequence. The predicted values of $X^{(0)}$ given by the GM(1,1) are:

$$\hat{x}_1^{(0)}(k+1) = (1 - e^a)[x^{(0)}(1) - \frac{b}{a}]e^{-ak}; \quad k = 1,2,\cdots,n. \tag{28}$$

Its residual series can be obtained as:

$$E^{(0)} = (\varepsilon^{(0)}(2), \varepsilon^{(0)}(3), \cdots, \varepsilon^{(0)}(n)), \tag{29}$$

where:

$$\varepsilon^{(0)}(k) = x^{(0)}(k) - \hat{x}^{(0)}(k); \quad k = 2,3,\cdots,n. \tag{30}$$

The residual can be expressed in the Fourier series as follows:

$$\hat{\varepsilon}_1^{(0)}(k) \cong \frac{1}{2}a_0 + \sum_{i=1}^{m}[a_i \cos(\frac{2\pi i}{T}k) + b_i \sin(\frac{2\pi i}{T}k)]; \quad k = 2,3,\cdots,n, \tag{31}$$

where $T = n - 1$, $m = \left\lfloor (\frac{n-1}{2}) \right\rfloor - 1$.

Rearrange Eq. (19) in matrix form as:

$$\hat{E}^{(0)} \cong PC, \tag{32}$$

where P and C matrixes can be defined as follows

$$P = \begin{bmatrix} 1/2 & \cos(2\frac{2\pi}{T}) & \sin(2\frac{2\pi}{T}) & \cos(2\frac{2\pi 2}{T}) & \sin(2\frac{2\pi 2}{T}) & \cdots & \cos(2\frac{2\pi m}{T}) & \sin(2\frac{2\pi m}{T}) \\ 1/2 & \cos(3\frac{2\pi}{T}) & \sin(3\frac{2\pi}{T}) & \cos(3\frac{2\pi 2}{T}) & \sin(3\frac{2\pi 2}{T}) & \cdots & \cos(3\frac{2\pi m}{T}) & \sin(3\frac{2\pi m}{T}) \\ \cdots & \cdots & \cdots & \cdots & \cdots & \cdots & \cdots & \cdots \\ 1/2 & \cos(n\frac{2\pi}{T}) & \sin(n\frac{2\pi}{T}) & \cos(n\frac{2\pi 2}{T}) & \sin(n\frac{2\pi 2}{T}) & \cdots & \cos(n\frac{2\pi m}{T}) & \sin(n\frac{2\pi m}{T}) \end{bmatrix} \quad (33)$$

$$C = \begin{bmatrix} a_0 & a_1 & b_1 & a_2 & b_2 & \cdots & a_m & b_m \end{bmatrix}^T \quad (34)$$

One can use the least-squares method to solve the Eq. (20) and calculate the matrix C as:

$$\hat{C} = (P^T P)^{-1} P^T E^{(0)} \quad (35)$$

The Fourier series correction can be obtained as follows:

$$\hat{x}_2^{(0)}(k) = \hat{x}_1^{(0)}(k) + \hat{\varepsilon}^{(0)}(k), \quad k = 2,3,\cdots,n. \quad (36)$$

References

1. Huang, K.Y.; Jane, C.J. A hybrid model for stock market forecasting and portfolio selection based on ARX, grey system and RS theories. *Expert Syst. Appl.* **2009**, *36*, 5387–5392.
2. Cui, J.; Liu, S.F.; Zeng, B.; Xie, N.M. A novel grey forecasting model and its optimization. *Appl. Math. Model.* **2013**, *37*, 4399–4406.
3. Hsu, C.C.; Chen, C.Y. Applications of improved grey prediction model for power demand forecasting. *Energ. Convers. Manage.* **2003**, *44*, 2241–2249.
4. Lin, Y.H.; Lee, P.C.; Chang, T.P. Adaptive and high-precision grey forecasting model. *Expert Syst. Appl.* **2009**, *36*, 9658–9662.
5. Xie, N.M.; Liu, S.F. Discrete grey forecasting model and its optimization. *Appl. Math. Model* **2009**, *33*, 1173–1186.
6. Kayacan, E.; Ulutas, B.; Kaynak, O. Grey system theory-based models in time series prediction. *Expert Syst. Appl.* **2010**, *37*, 1784–1789.

© 2017 by the authors; licensee MDPI, Basel, Switzerland. This article is an open access article distributed under the terms and conditions of the Creative Commons by Attribution (CC-BY) license (http://creativecommons.org/licenses/by/4.0/).
